# Computational Insights into Cyclodextrin Inclusion Complexes with the Organophosphorus Flame Retardant DOPO

**DOI:** 10.3390/molecules29102244

**Published:** 2024-05-10

**Authors:** Le Ma, Yongguang Zhang, Puyu Zhang, Haiyang Zhang

**Affiliations:** Department of Biological Science and Engineering, School of Chemistry and Biological Engineering, University of Science and Technology Beijing, Beijing 100083, China

**Keywords:** cyclodextrin inclusion complexes, organophosphorus flame retardants, molecular dynamics simulation, energy decomposition analysis

## Abstract

Cyclodextrins (CDs) were used as green char promoters in the formulation of organophosphorus flame retardants (OPFRs) for polymeric materials, and they could reduce the amount of usage of OPFRs and their release into the environment by forming [host:guest] inclusion complexes with them. Here, we report a systematic study on the inclusion complexes of natural CDs (*α*-, *β*-, and *γ*-CD) with a representative OPFR of DOPO using computational methods of molecular docking, molecular dynamics (MD) simulations, and quantum mechanical (QM) calculations. The binding modes and energetics of [host:guest] inclusion complexes were analyzed in details. *α*-CD was not able to form a complete inclusion complex with DOPO, and the center of mass distance [host:guest] distance amounted to 4–5 Å. *β*-CD and *γ*-CD allowed for a deep insertion of DOPO into their hydrophobic cavities, and DOPO was able to frequently change its orientation within the *γ*-CD cavity. The energy decomposition analysis based on the dispersion-corrected density functional theory (sobEDAw) indicated that electrostatic, orbital, and dispersion contributions favored [host:guest] complexation, while the exchange–repulsion term showed the opposite. This work provides an in-depth understanding of using CD inclusion complexes in OPFRs formulations.

## 1. Introduction

Flame retardants are extensively used in the flame-retardancy of polymeric materials. Halogenated flame retardants (HFRs) inhibited combustion reactions in the gas phase in most cases [1], thereby releasing toxic halogenated gases and having an impact on the environment [2,3] and organisms [4,5,6,7]. Compared to the HFRs, organophosphorus flame retardants (OPFRs) were reported to have a combined gas-phase and condensed-phase activity [8,9]. In the condensed phase, phosphoric acid substances generated from the combustion of OPFRs promoted the formation of a char layer on the materials. This char layer acted as a barrier to prevent the spread of flames and enhancing flame retardancy [10]. In the gas phase, the decomposition of OPFRs at high temperatures led to a release of phosphorus-containing radicals, which were able to capture the free radicals generated during combustion and interrupt the flame combustion process [11]. In light of the dual-phase flame-retardant mechanism, enhanced flame retardation, and low environmental impact [12], OPFRs have emerged as promising alternatives for flame retardancy.

Typical OPFRs include triphenyl phosphate (TPP), triethylphosphate (TEP), ammonium polyphosphate (APP), resorcinol bisdiphenylphosphate (RDP), and 9,10-dihydro-9-oxa-10-phosphaphenanthrene-10-oxide (DOPO). Most of them are additive flame retardants that do not form chemical bonds with polymers. To reach the desired flame retardancy effect, high loading is necessary [8]. As a consequence, the flame retardants had more of a chance to release into the environment via volatilization, leaching, and abrasion under production, transportation, application and disposal conditions [13,14,15]. Organophosphate esters (OPEs) have already been detected in indoor and outdoor air and dust [16,17,18,19], wastewater [20,21], soil [22,23], fish [24,25], and so on. Recently, cyclodextrins (CDs) and their derivatives have been introduced into the formulation of OPFRs because of enhanced flame retardancy performances and their ability to reduce the release of flame retardants via forming inclusion complexes with them [26,27,28,29,30,31,32].

**Figure 1:**
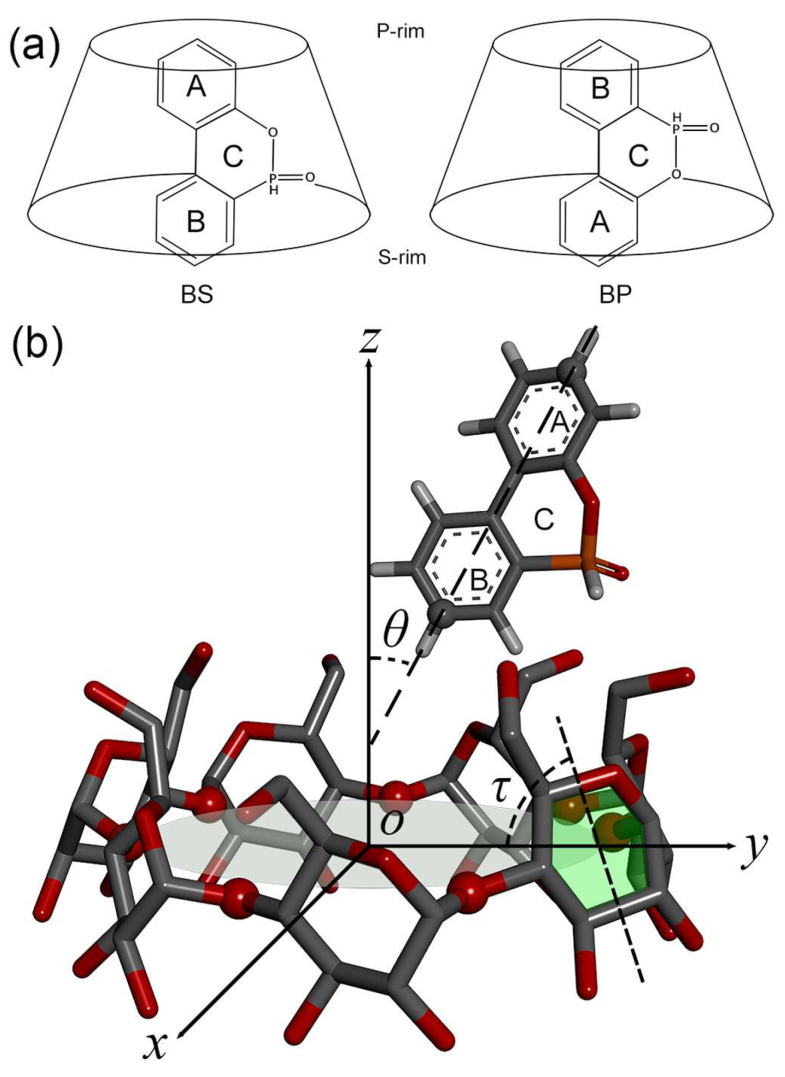
(**a**) Molecular structure of DOPO and its complexes with cyclodextrins (CDs) in the BS and BP binding modes. A, B, and C denote the ring groups in the guest DOPO. CDs are represented by truncated cones. BS means that the B ring of the guest is close to the secondary rim (S-rim) of CDs; BP refers to a binding pose with the B ring of the guest close to the primary rim (P-rim) of CDs. (**b**) The guest orientation of DOPO (*θ*) relative to the host *β*-cyclodextrin and the tilt angle (*τ*) of the glucose unit of CDs. The center of mass (COM) of glycosidic oxygen atoms (red balls) of CDs was set to the origin (*O*). The intersection angle (*θ*) between the vector connecting the two atoms (gray balls) of the guest and the normal vector (*Z*-axis) of the plane (*X-Y*, the gray ellipse) containing the glycosidic oxygen atoms is used to depict the guest orientation. The tilt angle (*τ*) between the plane (green) containing the pyran ring and the *X-Y* plane is used to describe the structural flexibility of the glucose unit in the CDs.

Cyclodextrins (CDs) are cyclic oligosaccharides obtained through the enzymatic degradation of linear starches [33], and were first discovered by Villiers in 1891 [34]. The common natural CDs are *α*-CD, *β*-CD, and *γ*-CD with 6, 7, and 8 D-glucopyranose units, respectively. They have a truncated cone structure with a hollow cone cavity [35], and the size and dimension of the cavity differs with the number of glucose units in CDs. The interior of the cavity is hydrophobic, composed of hydrogen atoms of C-3 and C-5 and oxygen atoms of glycosidic linkage, while the exterior is hydrophilic due to the presence of a large number of hydroxyl groups at primary and secondary rims [36], as shown in Figure 1 for *β*-CD. The hydrophobic cavity can accommodate a wide range of guest molecules to form noncovalent [host:guest] inclusion complexes (ICs). ICs typically serve as a beneficial modification to the physico-chemical properties of guest molecules. Therefore, CDs have been used in a variety of fields such as environment protection [37], pharmaceuticals [38], and flame retardancy [26].

CDs and their derivatives possess a huge number of hydroxyls, which can improve flame retardancy through the charring effect [39]. The [host:guest] ICs formed with the flame-retardant component can increase the interaction between components, enhance the thermal stability of flame retardants, and enable an even distribution of flame retardants (FRs) in polymer matrices. This allows for an effective immobilization and a reduced load of flame retardants, and eliminates unnecessary FR agent loss during the shelf life of flame-retardant products [26]. The excellent performances of CDs in OPFRs have been reported in a number of publications, and they are able to form ICs with, for instance, TPP [27,40], RDP [41], N,N′-diamyl-p-phenylphosphonicdiamide (P-MA) [29], Antiblaze RD-1 [31], and N,N′-dibutyl-phosphate diamide (DBPDA) [28].

DOPO and DOPO derivatives have been widely employed as an important OPFR in epoxy resins [28,29,42,43,44,45,46,47]; the molecular structure of DOPO is shown in Figure 1a. Very recently, Park et al. [32] undertook an experimental study on the inclusion complexes of DOPO with natural CDs of *α*-CD, *β*-CD, and *γ*-CD, and indicated the difference in the yields of ICs. They also examined the flame retardancy of cellulose acetate butyrate fibers using *γ*-CD as an additive. A combined experimental and computational method helped explore the underling mechanism of [host:guest] complexes between CDs and FRs, and provided a better understanding of CD complexation with guest molecules [27,40]. Our understanding of the CD complexes with DOPO at a molecular level is, however, still quite limited.

Here we reported a systematic investigation of the inclusion complexation between natural CDs (*α*-CD, *β*-CD, and *γ*-CD) and DOPO using a variety of computational approaches such as molecular docking, molecular dynamics (MD) simulations, and quantum mechanical (QM) calculations. This work focuses on the structures and energetics of the [host:guest] inclusion complexes and provides an in-depth understanding of the use of CD inclusion complexes in organophosphorus flame retardants.

## 2. Results and Discussion

### 2.1. Molecular Docking Prediction

For mimicking the formation of inclusion complexes between host and guest, we performed a series of docking calculations with varied search spaces. We first rotated and translated natural CDs (*α*-CD, *β*-CD, and *γ*-CD; host molecule) such that the CD cavity was almost parallel to the *Z*-axis and that the center of mass (COM) of the host CDs located at the origin (0, 0, 0), as shown in Figure 1b.

**Figure 2:**
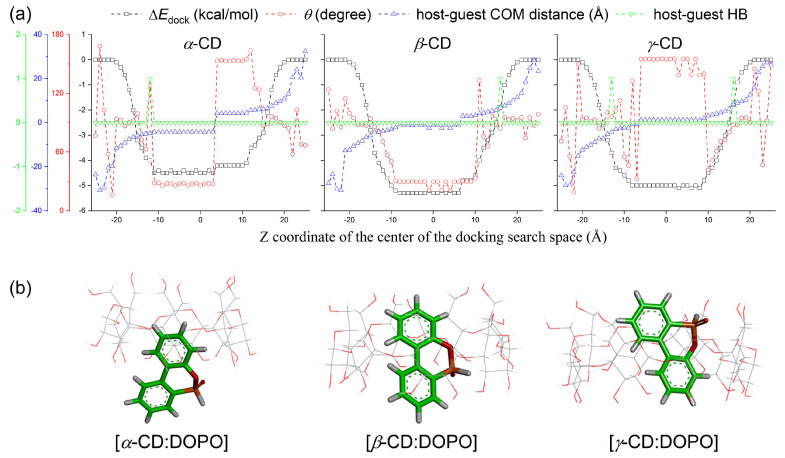
(**a**) Binding affinities (∆*E*_dock_, black squares) for [host:guest] complexation of CDs with DOPO as a function of the center *Z* of the docking search space. Host molecules were rotated and translated such that the CD cavity was almost parallel to the *Z*-axis (Figure 1b) and the center of mass (COM) of host CDs was positioned at the origin (0, 0, 0). Guest orientation was characterized by the angle (*θ*, red circles; see Figure 1b for the definition) and the COM distance (upward triangles in blue) between host and guest. A negative value for the distance indicates that the COM of the guest was much closer to the secondary rim (S-rim) of CDs than the primary rim (P-rim); a positive value means the guest COM was much closer to the P-rim (Figure 1). The number of hydrogen bonds (HB) between the host and guest is displayed with downward triangles in green. (**b**) The best binding modes of [host:guest] complexes from docking predictions. The CDs are displayed as line models, and the guest is given using stick models.

The center of the docking space was set to (0, 0, *z*), and *z* changed from −25 to 25 Å. As expected, [host:guest] COM distances changed from negative values to positive ones with the increasing *z*, as presented in Figure 2a. A negative distance indicates that the COM of the guest was much closer to the secondary rim (S-rim) of CDs than the primary rim (P-rim), and a positive distance means that the COM of the guest was close to the P-rim (for instance, Figure 1a). As *z* increased, the binding affinity (∆*E*_dock_) between host and guest started to increase, then reached a plateau, and finally decreased (Figure 2a). This implies that the guest DOPO was inserted into the CD cavity step by step, and then escaped from the cavity. At both ends of binding affinity profiles (Figure 2a), host and guest molecules were completely separated, and there was no interaction between them; the docking prediction gave a ∆*E*_dock_ of zero. The central plateau of binding affinity profiles correspond to the formed [host:guest] inclusion complexes. The plateau covered a range of ca. [−10, 10] Å because the box length of the docking search space was 20 Å and the height of the CD cone was 7.9 ± 1.0 Å [48]. In other words, if the search space covered the whole CD molecule, regardless of the given searching center, one could predict almost identical binding poses of the binding partners, as indicated by a significant span for the central plateau in Figure 2. Outside the central plateau, the guest did not penetrate into the CD cavity and was located outside the cavity in most cases, as indicated by the more positive ∆*E*_dock_ and an arbitrary guest orientation (*θ*) relative to CDs (Figure 2a).

The predicted binding poses derived from docking calculations are presented in Figure 2b, and the corresponding structural and energetic characterizations are listed in Table 1. The COM distance between *α*-CD and DOPO was −4.3 Å (Table 1), implying that a deep penetration of DOPO into the *α*-CD cavity was not allowed, and DOPO can only form a partial inclusion complex with *α*-CD, with its large portion residing outside the CD cavity (Figure 2b). On the contrary, it seemed easy for DOPO to enter into the hydrophobic cavities of *β*-CD and *γ*-CD with binding affinities of −5.3 and −5.0 kcal/mol and [host:guest] COM distances of −1.5 and 1.2 Å, respectively (Table 1).

In order to clearly depict the guest position inside the CD cavity, we defined a vector connecting two atoms in the ring groups of B and A of the guest and a normal vector (i.e., *Z*-axis) of the *X-Y* plane containing the glycosidic oxygen atoms, as shown in Figure 1b. The intersection angle (*θ*) of both vectors has been used to display the guest orientation relative to CDs. Such tasks can be performed using the GROMACS tool of “gmx gangle” [49]. The optimal binding modes for *α*-CD, *β*-CD, and *γ*-CD complexes with DOPO were predicted to be BS, BS, and BP, with guest orientations (*θ*) of 27.3, 28.1, and 151.8 degrees, respectively (Table 1). For *α*-CD, the docking also predicted BP modes with a binding affinity of ∆*E*_dock_ = −4.2 kcal/mol, a [host:guest] COM distance of 4.3 Å, and a guest orientation of *θ* = 153.4 degrees (Table 1). There were large jumps in ∆*E*_dock_, *θ*, and [host:guest] COM distance profiles between BP and BS binding modes for *α*-CD (Figure 2a), revealing that it was not feasible to form a complete inclusion complex between *α*-CD and DOPO. For *β*-CD, only the BS binding mode occurred. For *γ*-CD, the docking predicted a BS mode as well, yet with a small probability (Table 1).

The hydrogen bonds (HB) between host and guest molecules during the docking predictions indicate that DOPO was able to form one HB with all of three CDs (Figure 2a). However, The HB interaction only took place when DOPO stayed outside the CD cavity and no HB was observed for the best binding poses with strong binding affinities. This means that HB was likely not a driving force for [host:guest] associations.

### 2.2. Molecular Dynamics Simulations of Isolated and Complex Systems

#### 2.2.1. Structural Stability of Inclusion Complexes

Inclusion complexes of CDs with DOPO in the BS and BP binding modes (Figure 1a) were subjected to 100 ns MD simulations to monitor the complex stability. Each system was repeated three times (runs #1, #2, and #3) with different initial velocities. The [host:guest] COM distances obtained from the three replicas were combined together to obtain a distribution to determine whether the inclusion complexes were maintained during MD simulations or not, as presented in Figure S1 in the Supplementary Materials. For all of the simulated systems, there was a high peak with a [host:guest] COM distance of <6.5 Å (Figure S1); within such a distance, host and guest molecules formed an inclusion complex. For *α*-CD:DOPO and *γ*-CD:DOPO complexes using the BS binding mode as initial configurations, we also observed a small peak at 8.5 and 10 Å (Figure S1), respectively, where the inclusion complexes were disassociated and the guest was located outside the CD cavity. Therefore, we used a cutoff of 6.5 Å to discriminate the bound and unbound states.

For a clear characterization of guest position relative to host, the [host:guest] COM distances were set to positive or negative values, as discussed in the docking analysis (Section 2.1). For the bound states, the COM distances between guest and hydroxyl groups of CDs at P- or S-rims were calculated. If the distance between the guest and the P-rim of CDs was larger than that for the S-rim, the [host:guest] COM distance was set to a negative value. A positive distance meant that the COM of the guest was closer to the P-rim of CDs. For the unbound states, the COM distances were always positive.

**Figure 3:**
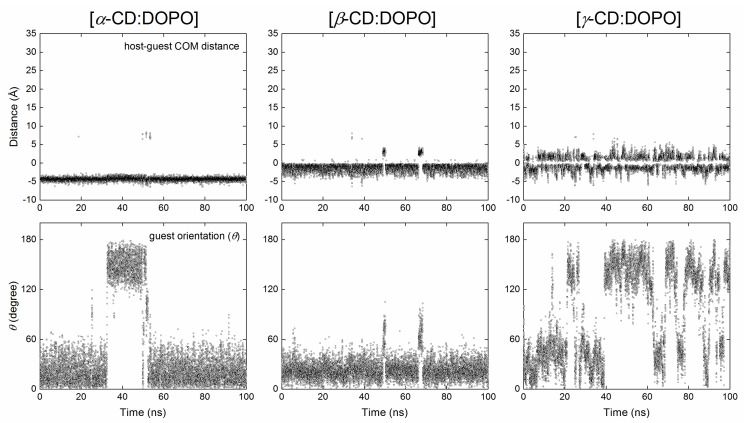
The center of mass (COM) distance between host and guest (**top**) and guest orientation (**bottom**) for the complexes of *α*-CD (**left**), *β*-CD (**middle**), and *γ*-CD (**right**) with DOPO as a function of simulation time using the BS binding mode (Figure 1a) as an initial configuration. A negative value for the distance indicates that the COM of the guest was much closer to the secondary rim (S-rim) of CDs than the primary rim (P-rim); a positive value means the guest COM was much closer to the P-rim (Figure 1). A distance of >6.5 Å indicates that the guest escaped from the CD cavity and did not form an inclusion complex with the host.

**Figure 4:**
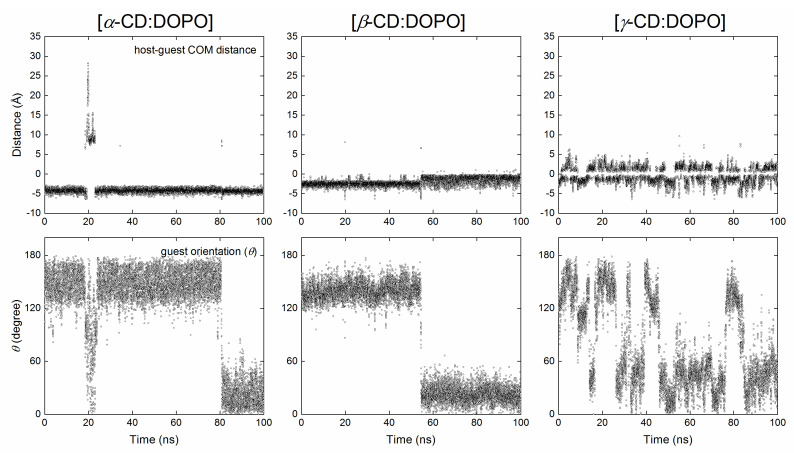
The center of mass (COM) distance between host and guest (**top**) and guest orientation (**bottom**) for the complexes of *α*-CD (**left**), *β*-CD (**middle**), and *γ*-CD (**right**) with DOPO as a function of simulation time using the BP binding mode (Figure 1a) as an initial configuration. Refer to the caption of Figure 3 for the meaning of positive and negative values for the [host:guest] distances.

[Host:guest] COM distance and guest orientation (*θ*) as a function of simulation time for the three replicas of MD simulations using BS and BP modes as initial configurations are given in the Supplementary Materials (Figure S2: *α*-CD for BS; Figure S3: *α*-CD for BP; Figure S4: *β*-CD for BS; Figure S5: *β*-CD for BP; Figure S6: *γ*-CD for BS; Figure S7: *γ*-CD for BP). Representative replicas are presented in Figure 3 and Figure 4 with initial configurations of BS and BP, respectively. According to the definition in Figure 1b, if host and guest molecules formed an inclusion complex in the BS binding mode, the angle *θ* should be smaller than 90 degrees; for the BP mode, *θ* should be larger than 90 degrees.

**Figure 5:**
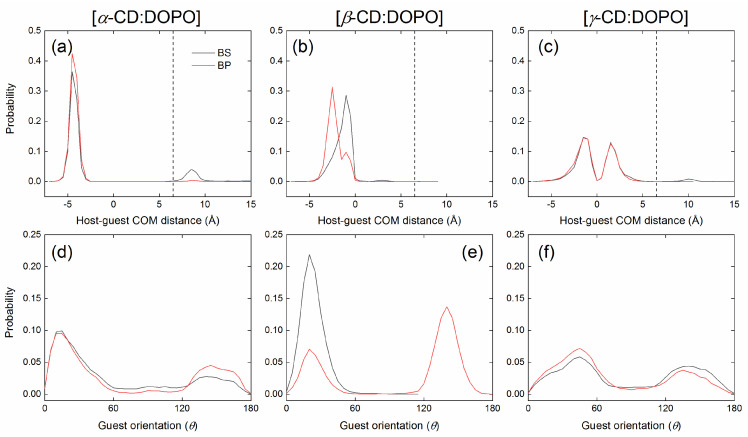
Distribution of the center of mass (COM) distance between host and guest (top, **a**–**c**) and guest orientation relative to host (bottom, **d**–**f**) for the complexes of *α*-CD (left), *β*-CD (middle), and *γ*-CD (right) with DOPO during MD simulations using BS and BP binding modes (Figure 1a) as initial configurations. The dash lines indicate a distance of 6.5 Å, which separates the binding pose of the guest included in the CD cavity from the pose of the guest located outside the CD cavity. Each system was simulated for 100 ns and repeated three times using different initial velocities. Refer to the caption of Figure 3 for the meaning of positive and negative values for the [host:guest] distances.

For *α*-CD complexes with DOPO, the guest was observed to escape from the *α*-CD cavity and return to the cavity again during MD simulations. As a result, the binding modes jumped between BS and BP (Figure 3 and Figure 4), independent of the initial configurations used for MD simulations. In some cases, the guest cannot enter the *α*-CD cavity once it has escaped, as indicated by a large [host:guest] COM distance and an arbitrary *θ* (run #2, Figure S2). DOPO preferred to stay close to the secondary rim (S-rim) of *α*-CD in the BS binding mode, with a [host:guest] COM distance of −4.5 Å (Figure 5a) and a guest orientation of *θ* = 13 degrees (Figure 5d). This is in line with the docking predictions. Unlike the docking results, however, the COM of the guest in the BP mode was closer to the S-rim than the P-rim (Figure 2a and Figure 5a).

A jump between binding modes of BS and BP was observed for *β*-CD complexes with DOPO as well. It appears that the jump for *β*-CD complexes was not as frequent as it was for *α*-CD complexes (Figure 3 and Figure 4). The COM of DOPO in the BS binding mode appeared to be closer to the COM of *β*-CD than did that in the BP mode (Figure 4, Figure 5, and Figure S5). When using the host of *β*-CD, DOPO preferred to stay close to its secondary rim (S-rim) in the BS binding mode with a [host:guest] COM distance of −1.0 Å (Figure 5b) and a guest orientation of *θ* = 20 degree (Figure 5e). This finding agrees with the docking predictions. We have also observed the BP binding mode with a relatively high probability, which was not, however, detected in the docking. High peaks in the distribution of guest orientation *θ* (Figure 5e) indicate that the guest DOPO cannot easily escape from the cavity and change its orientation within the *β*-CD cavity once the inclusion complexes were formed.

The jump of binding modes between BS and BP was very frequent when the guest DOPO was in complex with *γ*-CD (Figure 3, Figure 4, Figures S6 and S7). BS and BP modes seem to exist with an equal probability, and the BS mode was slightly preferred over the BP mode, as indicated by a slightly higher probability of guest orientation at *θ* = 45 degrees than that at *θ* = 140 degrees (Figure 5c,f). This implies that DOPO can easily insert into or escape from the *γ*-CD cavity.

#### 2.2.2. Tilt Angle of Glucose Units with and without Guest inside the CD Cavity

**Figure 6:**
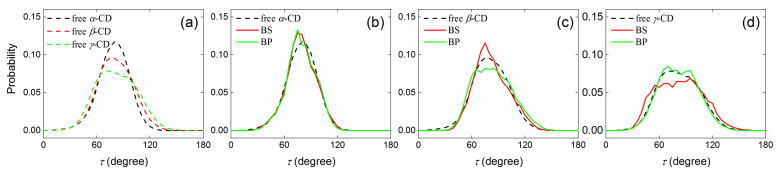
Distribution of the tilt angle (*τ*) of the glucose units during MD simulations of the free (isolated) hosts (**a**) and [host:guest] inclusion complexes for *α*-CD (**b**), *β*-CD (**c**), and *γ*-CD (**d**). The dashed lines indicate the systems of free CDs, and the solid lines are for the inclusion complexes of CDs with DOPO in the binding modes of BS and BP.

The tilt angle (*τ*) was a measure of the overall shape of CDs, and a smaller *τ* corresponds to a more open S-rim of CDs and a narrower P-rim. During MD simulations of isolated (free) CDs without a guest, the tilt angle (*τ*) amounted to ca. 80 degrees (Figure 6a). The distribution of *τ* for *α*-CD was the narrowest, while *γ*-CD showed the widest distribution (Figure 6a). This indicates that the structural rigidity of CDs were in the order of *α*-CD > *β*-CD > *γ*-CD.

Upon complexation with the guest DOPO, the *α*-CD structure became more rigid, as indicated by a higher peak than that without guest, and the tilt angle *τ* was shifted to smaller values (Figure 6b). This can be ascribed to the fact that DOPO preferred to stay close to the secondary rim (S-rim) of *α*-CD (Figure 2b and Figure 5a), thereby resulting in a more open S-rim (that is, a smaller *τ*). Similar to *α*-CD, DOPO tended to reside within the *β*-CD cavity with a negative [host:guest] COM distance (that is, close to S-rim), and such binding led to a much narrower distribution for the BS mode (red lines in Figure 6c). Because of the jump of binding modes and the small [host:guest] COM distance, the structure of *β*-CD in the BP binding mode appeared more flexible than that in the BS mode, as indicated by a lower and wider distribution (green lines in Figure 6c). Compared to the free *β*-CD, there was no significant shift in the peaks of the tilt angle *τ* for *β*-CD complexes with the guest, indicating that the formation of a complete inclusion complex with the guest DOPO helped maintain the overall shape of the host *β*-CD. Similarly, no significant changes for the tilt angle *τ* were observed for *γ*-CD complexes with DOPO, and the BS binding mode appeared to yield a more flexible *γ*-CD than the BP mode (Figure 6d). These structural changes upon complexation of the binding partners imply that the host molecule can be rearranged to fit the guest and vice versa, also known as the “induced-fit” effect [50].

### 2.3. Binding Free Energy Calculations via MM–PBSA

Binding energies (Δ*E*_bind_) between host and guest molecules were computed by the MM–PBSA analysis using the MD simulation trajectories with the aid of the “gmx_MMPBSA” toolkit [51]. There occurred jumps between the binding modes of BS and BP during 100 ns simulations (mentioned above), and we therefore chose a 10 ns-long trajectory in which the binding modes were kept unchanged, and extracted 1001 frames with a step of 10 ps for the MM–PBSA analysis.

Δ*E*_bind_ can be further decomposed into MM and solvation contributions (refer to Equations (1)–(3) in Section 3.3), and the results are tabulated in Table 2. The bonded interactions (Δ*E*_bonded_) due to the “induced-fit” effect disfavored [host:guest] associations with a contribution of 1–3 kcal/mol. Because DOPO is a relatively rigid molecule (Figure 1), it contributes little to the complexation, and most of the bonded interactions came from the host CDs (Table 2). The nonbonded van der Waals (∆*E*_vdW_) and electrostatic (∆*E*_elec_) interactions also made a favorable contribution, and ∆*E*_vdW_ contributed ca. 70–85% of the nonbonded interactions (Δ***E***_nonbonded_).

The solvation part (Δ*G*_sol_) disfavored the binding (Table 2), likely due to the desolvation effect upon association of the binding partners. From the total binding energies (Δ*E*_bind_), it was concluded that the BP binding mode was more stable for *α*-CD and *γ*-CD complexes than BS, whereas the BS mode was more stable for *β*-CD complexes.

MD trajectories were also used for the MM–PBSA analysis to analyze the [host:guest] HB interactions via the GROMACS tool of “gmx hbond” [49]. During the MD simulations, the average number of HBs between CDs and DOPO was smaller than one, and it appeared that the BP modes provided more HB contacts than the BS mode (Table S1).

### 2.4. QM-Optimized Structures

**Figure 7:**
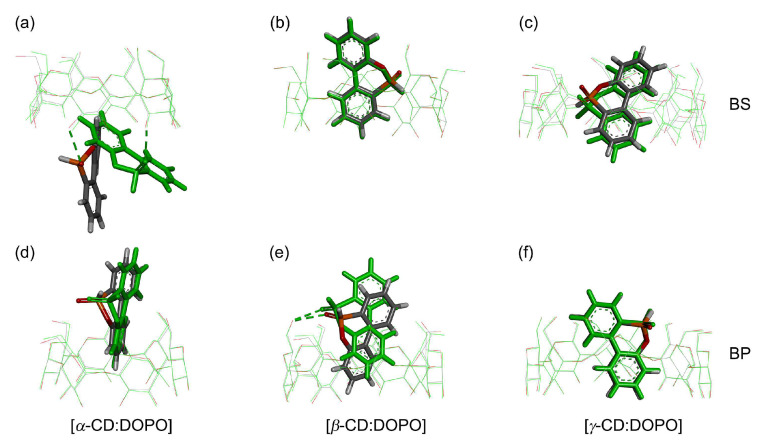
QM-optimized [host:guest] complexes at B3LYP/6-31G** in gas (green) and water (colored by elements) phases for the binding modes of BS (top, **a**–**c**) and BP (bottom, **d**–**f**). Host molecules are displayed with line models, and guest models are shown with stick models. Green dashed lines reveal the [host:guest] hydrogen bonds. For a comparison of guest poses in different phases, the structures of CDs in water are superimposed on those in gas via the least-squares fitting of non-hydrogen atoms of glucose pyran rings.

Optimized complex structures in gas and water phases are presented in Figure 7, and the corresponding structural properties are listed in Table 3. DOPO formed one HB with hydroxyl groups of *α*-CD and *β*-CD in the binding mode of BS and BP, respectively. In line with the docking predictions, *α*-CD and DOPO were not able to form a complete inclusion complex in the gas phase, and a large portion of the guest was located outside the *α*-CD cavity with a [host:guest] COM distance of −5.2 and 4.5 Å for the BS (Figure 7a) and BP (Figure 7d) binding modes, respectively. The COM of DOPO in the binding BP mode was close to the primary rim (P-rim) of *α*-CD in the docking and QM predictions (Figure 7d), whereas MD simulations show that the COM of DOPO tended to stay in the secondary rim (S-rim) of *α*-CD (Figure 5a). Compared to the optimized BS poses in the gas phase, there was a big difference in the guest orientation, which changed from 57.1 to 31.1 degrees, and the COM of the guest was a little further away from the *α*-CD cavity (Table 3). No significant differences in the BP binding mode were observed for the *α*-CD:DOPO complexes in gas and water phases.

QM calculations show that the COMs of DOPO were close to the P-rim of *β*-CD, with [host:guest] COM distances of 1.5 and 3.8 Å for the BS (Figure 7b) and BP (Figure 7e) modes, respectively. This is dramatically different from the results of docking and MD simulations, in which the COM of DOPO was found to be close to the S-rim of *β*-CD (Figure 2 and Figure 5b). The COM of the guest appeared to stay closer to the *β*-CD cavity in the BP mode in water than in the gas phase (Figure 7e and Table 3).

Similar to *β*-CD, complete inclusion complexes were formed between *γ*-CD and DOPO, and the COM of the guest was roughly located at the COM of *γ*-CD, as indicated by the small [host:guest] COM distances (Figure 3, panel right; Table 3). No obvious changes were observed for the optimized structures in gas and water phases except for a small shift from −0.1 Å (gas) to 0.6 Å (water) in the [host:guest] COM distances for the BS binding mode.

We also calculated the tilt angle (*τ*, Figure 1b) of glucose units and circularity to characterize the overall shapes of CDs. In the crystal structure without a guest, the tilt angles (*τ*) were 85.2, 84.5, and 83.8 degrees for *α*-CD, *β*-CD, and *γ*-CD, respectively (Table 3). Compared to the crystal structures, QM optimization yielded smaller *τ* values of 78.8, 81.7, and 79.5 degrees for *α*-CD, *β*-CD, and *γ*-CD, respectively (Table 3). That is, QM calculations predicted a narrower P-rim and a more open S-rim of CDs, in particular for *α*-CD. The tilt angle of glucose units (*τ*) seemed to be sensitive to the binding position of guest molecules. For instance, DOPO was located close to the P-rim of *α*-CD in the BP mode (Figure 7d), in which the tilt angle amounted to 88 degrees, larger than the case without guest (78.8 degree) and the BS mode with the guest close to the S-rim (77.5 degrees). Compared to the *β*-CD:DOPO complex in the BP mode in gas (*τ* = 80.7), a deeper insertion into the *β*-CD cavity in water led to a larger tile angle of 83.1 degrees (Table 3).

Circularity is a distortion parameter of the CD cavity [52]. A value of 1 indicates a perfectly circular shape, and a small circularity implies that the CD cavity was compressed or stretched due to, for instance, guest binding, and hence changed to an elliptical shape [53], as observed in our previous work [54]. In this work, binding with DOPO affected the shape of the CD cavity as well, in particular for the *γ*-CD:DOPO complexes in which the guest displayed a deep insertion into the *γ*-CD cavity (Table 3). For the BS mode in gas, the circularity changed from 0.954 (without guest) to 0.912 (with guest) for the *γ*-CD complexes with DOPO, whereas no significant changes were observed for the BP mode. These changes in the tilt angle of glucose units and circularity were related to the “induced-fit” effect mentioned above, implying that the host CDs underwent conformational changes upon association with guest molecules.

### 2.5. Energy Decomposition Analysis (EDA)

#### 2.5.1. EDA-FF

The energy decomposition analyses based on the force field (EDA-FF) [55] were performed using the QM-optimized inclusion complexes of CDs with DOPO. Interaction energy between host and guest (Δ***E***_FF_, kcal/mol) in the EDA-FF framework was a sum of electrostatic (Δ***E***_elec_), repulsion (Δ***E***_rep_), and dispersion (Δ***E***_disp_) contributions (refer to Equation (4) in Section 3.5.1). Different electrostatic potential (ESP) fitting methods for deriving atomic charges were examined, namely, Merz–Kollmann (MK), CHELPG, RESP, and RESP2.

The EDA-FF results based on the Amber force field indicate that Δ***E***_elec_ and Δ***E***_disp_ were negative and favored [host:guest] binding, whereas Δ***E***_rep_ showed the opposite (Table 4). The BS binding mode was more stable than the BP mode for *α*-CD and *γ*-CD complexes with DOPO, while for *β*-CD:DOPO complexes, BP was more stable than BS. This finding is independent of the choice of the charge methods. The stability of inclusion complexes was on the order of *β*-CD > *α*-CD > *γ*-CD for the MK, CHELPG, RESP, and RESP2 charges. However, the order was changed to *β*-CD > *γ*-CD > *α*-CD when using the atomic charges from the used force fields in MD simulations (FFMD), in line with the Δ***E***_MM_ in the MM–PBSA analysis (Table 2). The result of EDA-FF is, by nature, very similar to the [host:guest] interaction energy obtained via MD simulations, and it is also useful and often used to judge the binding affinities between host and guest molecules for comparison with experimental observations. This highlights the importance of the choice of charge methods in the evaluation of [host:guest] interactions. Note that RESP and RESP2 charges have been recommended for use with Amber force fields, and the FFMD charge was Amber-like as well.

In most cases, the RESP2 charge produced more negative Δ***E***_elec_ and Δ***E***_FF_. This might be due to the consideration of charge distribution in both gas and water phases in the RESP2 protocol [56]. Compared to the gas-phase-optimized structures, using the water-phase-optimized complexes gave a relatively weak binding affinity, as indicated by the more positive energies in Table 3. This can likely be ascribed to the screening effect of solvent media.

#### 2.5.2. sobEDAw

The energy decomposition analyses based on the dispersion-corrected density functional theory (sobEDA) [57] were performed using the QM-optimized inclusion complexes of CDs with DOPO. We also used the optimized structurers of isolated host and guest molecules to consider the effects of geometry deformation (Δ*E*_def_) and solvation (Δ*E*_sol_) as well as the thermal correction (Δ*G*_corr_). For a direct comparison with the popular symmetry-adapted perturbation theory (SAPT) [58], we used a variant of sobEDA, specifically for the decomposition of weak interaction energies (denoted as sobEDAw) [57]. For our calculations with the B3LYP-D3(BJ)/6-31+G(d,p) level of theory, about 59% of the DFT correlation energy (Δ*E*_DFTc_) was combined with the dispersion correction (Δ*E*_dc_), the resulting one (Δ*E*_disp_) being an SAPT-like dispersion term (Equation (8) in Section 3.5.2).

The interaction energy (Δ*E*_int_) consists of electrostatic (∆*E*_els_), orbital interaction (∆*E*_orb_), dispersion (Δ*E*_disp_), and exchange-repulsion (∆*E*_xrep_). For all of the CD complexes with DOPO, the first three terms favored the binding, while the last one did not (Table 5). In agreement with the bonded interactions (Δ*E*_bonded_) from the MM–PBSA analysis, Δ*E*_def_ disfavored binding. This finding indicates that the host would adjust its conformations for a good fit to maximize nonbonded interactions with guest, which likely came at the cost of an increase in the bonded energies of the binding partners. Δ*E*_sol_ and Δ*G*_corr_ displayed unfavorable contributions as well.

Interestingly, interaction energies between CDs and DOPO from the predictions of MM–PBSA (Δ***E***_MM_, Table 2), EDA-FF (Δ***E***_FF_, Table 4), and sobEDAw (Δ***E***_MM_, Table 5) were in the same order of magnitude. Note that Δ***E***_FF_ was, in nature, similar to the nonbonded interactions (Δ*E*_nonbonded_) in Δ***E***_MM_, and both terms were computed using identical potential functions. This finding reveals that the force field-based evaluations were comparable in their results to the expensive DFT calculations.

From the interaction energies (Δ*E*_int_), we can see that *β*-CD complexes were the most stable, followed by *γ*-CD and *α*-CD complexes (Table 5). However, *α*-CD and *β*-CD complexes underwent a relatively large deformation in their structures, which resulted in a large contribution disfavoring binding. As a result, *γ*-CD complexes with DOPO appeared thermodynamically favorable (negative Δ*G*_bind_, Table 5). This agrees with the experiments by Park et al., where they did not detect the yield of *α*-CD inclusion complexes with DOPO, and the yield of *γ*-CD complexes was greater than that of *β*-CD [32]. For the optimized structures in the gas phase, the sobEDAw analysis gave positive Δ*G*_bind_ for all of the inclusion complexes, highlighting the importance of a solvation environment in the complexation of CDs with DOPO.

## 3. Materials and Methods

### 3.1. Molecular Docking

Molecular structures of natural CDs (host molecules) were extracted from the Protein Data Bank (PDB IDs: 1CXF [59] for *α*-CD, 3CGT [60] for *β*-CD, and 1D3C [61] for *γ*-CD). The 3D structure of DOPO (guest) was obtained from the Pubchem Substance and Compound Databases [62]. Hydrogen atoms of CDs were added using the Biovia Discovery Studio visualization software (version 2019).

The Autodock Vina software (version 1.1.2) [63] was utilized to perform docking predictions of the binding poses between host and guest molecules. CDs were rotated and translated, making the CD cavity almost parallel to the *Z*-axis and their center of mass (COM) locating at the origin (0, 0, 0), as shown in Figure 1b. This can be done using the GROMACS (version 2018.4) [49] tool of “gmx editconf”. The search space was 20 × 20 × 20 Å^3^ with a center of *x* = 0, *y* = 0, and *z* ranging from −25 to 25 Å with a step of 1 Å. That is, we performed 51 docking calculations in total for each cyclodextrin, mimicking the formation process of [host:guest] complexes. As the center *z* increases, the guest DOPO enters into the CD cavity from a distance far away from the host and escapes from the cavity then. In the docking, CD structures were kept fixed, while DOPO was treated as flexible. The Vina scoring [63] was used to evaluate the sampled conformations of [host:guest] associations. Such scoring was trained using the experimentally measured binding affinities of protein–ligand complexes with known 3D structures under a condition closest to room temperature and neutral pH, as collected in the PDBbind database [64,65]. The binding poses with the strongest binding affinities were saved for data analysis. Other docking parameters were set by default.

### 3.2. Molecular Dynamics Simulation

The q4md-CD force field [66], specific to CD-containing systems, was chosen to model natural CDs. It was based on the GLYCAM04 [67,68,69] and Amber99SB [70] force fields and therefore belonged to the Amber-like force field family. Force field parameters of *α*-CD, *β*-CD, and *γ*-CD were taken from our previous work [71] and have been deposited in the website https://virtualchemistry.org/ff.php (accessed on 11 April 2024). The General Amber Force Field (GAFF2) [72] was adopted to model the guest DOPO. The guest structure was optimized at HF/6-31G* in the gas phase via the Gaussian 16 software (Revision A.03) [73] and we then computed its restrained electrostatic potential (RESP) charges.

Similar to the preparation of host structures in the docking (Section 3.1), DOPO was also rotated to make its longest axis parallel to the *Z*-axis and locate its COM at the origin (0, 0, 0). The combination of host and guest structures together would then give rise to inclusion complexes between CDs and DOPO. Two binding modes of BS and BP were examined in this work, as shown in Figure 1a. BS indicates that the B ring of the guest was much closer to the secondary rim (S-rim) than the primary rim (P-rim); BP stands for a binding pose with the B ring of the guest close to the P-rim of CDs.

The [host:guest] inclusion complex was regarded as a solute and was placed in a cubic box where the distance between the box edge and solute was set to 12 Å. The simulation box was then filled with a pre-equilibrated water box; as a result, 1600, 1760, and 1900 water molecules were inserted into the simulation cell for the *α*-CD, *β*-CD, and *γ*-CD complexes, respectively. Each system was simulated for 100 ns and was repeated three times with different initial velocities. Similarly, MD simulations of isolated hosts for *α*-CD, *β*-CD, and *γ*-CD were also extended to 100 ns for a comparison with the cases in the presence of a guest. All of the MD simulations were carried out using the GROMACS (version 2018.4) [49] software at *NPT* ensemble (*P* = 1 bar and *T* = 298.15 K). The details for the MD protocol are presented in our previous reports [71,74,75,76].

### 3.3. MM–PBSA Analysis

[Host:guest] binding free energies were calculated via molecular mechanics Poisson–Boltzmann surface area (MM–PBSA) analysis using the “gmx_MMPBSA” package [51,77]. After removing water molecules, 1001 complex frames were extracted from the MD trajectories and were used for the analysis.

Binding free energy (∆*G*_bind_) upon [host:guest] complexation is composed of the molecular mechanics (MM, ∆*E*_MM_), solvation (∆*G*_sol_), and entropy (∆*S*) parts (Equation (1)).

∆*G*_bind_ = ∆*E*_MM_ + ∆*G*_sol_ − *T*∆S (1)

∆*E*_MM_ can be further decomposed into bonded (∆*E*_bonded_) and nonbonded (∆*E*_nonbonded_) contributions, and ∆*E*_nonbonded_ is a sum of van der Waals (∆*E*_vdW_) and electrostatic (∆*E*_elec_) interactions (Equation (2)) [75,76,78].

∆*E*_MM_ = ∆*E*_bonded_ + ∆*E*_nonbonded_ = ∆*E*_bonded_ + ∆*E*_vdW_ + ∆*E*_elec_ (2)

The solvation part (∆*G*_sol_) can be decomposed into polar (∆*G*_polar_) and nonpolar (∆*G*_nonpolar_) contributions (Equation (3)).

∆*G*_sol_ = ∆*G*_polar_ + ∆*G*_nonpolar_ (3)

In practical applications, entropy (∆S) calculation requires a heavy computational load and its evaluation strongly depends on the used methods. Therefore, we chose not to calculate the entropy contribution in this work, and considered only the binding energy (∆*E*_bind_ = ∆*E*_MM_ + ∆*G*_sol_).

### 3.4. QM-Optimized Structures of Inclusion Complexes

The [host:guest] inclusion complexes and isolated host and guest molecules prepared for MD simulations (Section 3.2) were used as initial configurations for QM optimization at the B3LYP/6-31G** level of theory in the gas phase using the Gaussian 16 (Revision A.03) [73] software. The optimized structure in gas was then used as a starting point for subsequent optimization in the water phase. The integral equation formalism variant of polarizable continuum model (IEFPCM) was adopted to describe the water medium [79]. Frequency calculations were performed after optimization to ensure the convergence of QM optimization without imaginary frequency.

### 3.5. Energy Decomposition Analysis (EDA)

The EDA analysis was used to pinpoint individual contributions to the [host:guest] complexation, and such tasks were carried out using the Multiwfn (version: 3.8) [80] and Gaussian 16 (Revision A.03) [73] software. In this work, we examined two types of EDA analysis based on the force field (EDA-FF for short) [55] and on the dispersion-corrected density functional theory (denoted as sobEDA) [57].

#### 3.5.1. EDA-FF

Similar to the nonbonded contributions to the binding in the MM-PBSA analysis (Section 3.3), interaction energies (∆*E*_FF_) between fragments A and B in the EDA-FF framework were decomposed into two components of the electrostatic (∆*E*_elec_) and van der Waals (∆*E*_vdW_) forces. ∆*E*_elec_ was computed by the Coulomb law, and ∆*E*_vdW_ was modeled by the common Lennard–Jones (LJ) 12–6 potential (*C*_12_ term for repulsion, ∆*E*_rep_; *C*_6_ term for dispersion, ∆*E*_disp_), as given in Equation (4).

(4) $$\Delta E_{\mathrm{FF}}=\Delta E_{\mathrm{elec}}+\Delta E_{\mathrm{vdw}}=\Delta E_{\mathrm{elec}}+\Delta E_{\mathrm{rep}}+\Delta E_{\mathrm{disp}}=\underset{i\in A}{\sum }\underset{j\in B}{\sum }\left[\frac{1}{4\pi \varepsilon_{0}}\frac{Q_{i}Q_{j}}{r_{ij}}+\varepsilon_{ij}{\left(\frac{R_{\min,\;ij}}{r_{ij}}\right)}^{12}-\varepsilon_{ij}{\left(\frac{R_{\min,\;ij}}{r_{ij}}\right)}^{6}\right]$$

where *Q*_i_ and *Q*_j_ are partial charges of atoms *i* and *j*, *r*_ij_ is atom–pair distance, *ε*_0_ is vacuum permittivity, *ε_ij_* and *R*_min_ are the well depth of 12-6 LJ potential and the atom–pair distance at which the LJ potential reaches its minimum.

The atom types in the general Amber force field [72,81] (often given in a form of *ε_ij_* and *R*_min_/2) were used for the computation of ∆*E*_vdW_, and the atomic charges were derived using electrostatic potential (ESP) fitting methods [82,83] such as Merz–Kollmann (MK), CHELPG, RESP, and RESP2 [56]. Single-point energy calculations of the optimized isolated host and guest molecules were carried out at the B3LYP-D3(BJ)/def2-TZVP level of theory. MK, CHELPG, and RESP charges were computed in the gas phase, while atomic charges for RESP2 were averaged over the computed charges in gas and water phases; that is, a tuning factor of *δ* = 0.5 was used [56]. All of the charge calculations were performed with the Multiwfn (version: 3.8) [80] software using the built-in ESP evaluation algorithm [84].

#### 3.5.2. sobEDA

In the sobEDA framework, the interaction energy (∆*E*_int_) was composed of electrostatic (∆*E*_els_), exchange (∆*E*_x_), correlation (∆*E*_DFTc_), dispersion correction (∆*E*_dc_), Pauli repulsion (∆*E*_rep_), and orbital interaction (∆*E*_orb_) terms. ∆*E*_x_ and ∆*E*_rep_ can be merged together as the exchange–repulsion term (∆*E*_xrep_), as shown in Equation (5).

(5) $$\Delta E_{\mathrm{int}}=\Delta E_{\mathrm{els}}+\Delta E_{x}+\Delta E_{\mathrm{DFTc}}+\Delta E_{\mathrm{dc}}+\Delta E_{\mathrm{rep}}+\Delta E_{\mathrm{orb}}=\Delta E_{\mathrm{els}}+\Delta E_{\mathrm{xrep}}+\Delta E_{\mathrm{orb}}+\Delta E_{\mathrm{DFTc}}+\Delta E_{\mathrm{dc}}$$

Here we used a variant of sobEDA for weak interactions (denoted as sobEDAw). For a direct comparison with the popular symmetry-adapted perturbation theory (SAPT) method [58], Lu and Chen proposed a weighting factor ω to determine which portion of correlation energy (∆*E*_DFTc_) could be combined with the dispersion correction (∆*E*_dc_), resulting in a new dispersion term (∆*E*_disp_) comparable with SAPT, and the remaining can be incorporated into the exchange–repulsion term (∆*E*_xrep_), as given in Equations (6)–(8) [57].

(6) $$\Delta E_{\mathrm{xrep}}=\Delta E_{\mathrm{xrep}}^{\mathrm{sobEDA}}+\left(1-\omega \right)\Delta E_{\mathrm{DFTc}}$$

(7) $$\Delta E_{\mathrm{disp}}=\Delta E_{\mathrm{dc}}+\omega \Delta E_{\mathrm{DFTc}}$$

(8) $$\omega =\exp\left[-r\left(\frac{\Delta E_{\mathrm{dc}}}{\Delta E_{\mathrm{els}}}-a\right)\right]\left(1-c\right)+c$$

Note that the weight parameters in Equation (8) highly depend on the used level of theory during DFT calculations. As recommended, we used B3LYP-D3(BJ)/6-31+G(d,p) for the sobEDA analysis, for which *r* = 2.571, *a* = 0.071, and *c* = 0.575 [57].

Moreover, we also considered the effects of geometry deformation (Δ*E*_def_), solvation (Δ*E*_sol_), and thermodynamic free energy correction on the interaction energy (Δ*G*_corr_) upon complexation of the binding partners. Combining all of the energy terms together, we obtained the binding free energies (Δ*G*_bind,_ Equation (9)).

(9) $$\Delta G_{\mathrm{bind}}=\Delta E_{\mathrm{int}}+\Delta E_{\mathrm{def}}+\Delta E_{\mathrm{sol}}+\Delta G_{\mathrm{corr}}$$

We followed the protocols set out in the work by Lu and Chen [57] to perform the sobEDAw analysis. The SMD model was used to describe the solvation effect at M05-2X/6-31G*, a level of theory in line with the development and testing of this solvation model [85]. The Shermo package (version 2.4.1) [86] was used to extract the thermochemistry data from frequency calculations, and frequency scale factors were set to 0.9875, 1.0062, and 1.0099, in line with the used level of theory [87], for the calculation of zero-point energy, the contribution of heating to internal energy, and entropy, respectively.

## 4. Conclusions

In this work, we undertook a computational investigation of the complexation between natural CDs (*α*-CD, *β*-CD, and *γ*-CD; host) and the organophosphorus flame retardant DOPO (guest) via molecular docking, molecular dynamics (MD) simulations, and quantum mechanical (QM) approaches. QM calculations offered dramatically different results from docking and MD in the predicted binding poses and binding energies, which were mainly due to the differences in the treatment of electrons and solvent media. Despite these discrepancies using different methods, all of these methods indicated that *α*-CD was not able to form a complete inclusion complex with DOPO, and *β*-CD and *γ*-CD allowed for deep insertions of the guest into their cavities. On the basis of interaction energies, *β*-CD complexes appeared more stable than *γ*-CD and *α*-CD complexes. This means that the cavity size of *β*-CD matched well with that of DOPO. Due to a large cavity of *γ*-CD, it was very easy for DOPO to change its orientation within the *γ*-CD cavity. Considering the geometry deformation, solvation, and thermodynamic effects, the resulting binding free energies indicate that *γ*-CD complexes were the most stable and thermodynamically favorable. This agrees with the experimental measurements performed by Park et al., whereby they obtained a greater yield of *γ*-CD complexes than *β*-CD and no yield of *α*-CD complexes with DOPO [32]. They also proposed a binding mode of *γ*-CD with DOPO, in which only the aromatic ring (ring A in Figure 1) penetrated into the *γ*-CD cavity (that is, forming a partial inclusion complex), based on the ^1^H NMR results in a solution of DMSO. This is in opposition to our MD simulations and QM calculations, probably due to the difference in the solvent medium used. This work provides implications for CD complexes with DOPO, and is valuable in relation to the potential applications of CDs in the formulations of organophosphorus flame retardants.

## Figures and Tables

**Table 1:** Predicted binding poses between cyclodextrins (CDs) and DOPO by docking calculations.

Cyclodextrin	Interval of Center *Z* (Å) *^a^*	∆*E*_dock_ (kcal/mol) *^b^*	*θ* (degree) *^c^*	COM Distance (Å) *^d^*	Binding Mode *^e^*
*α*-CD	[−11, 3]	−4.5	27.3 ± 1.0	−4.3 ± 0.0	BS
*α*-CD	[4, 10]	−4.2	153.4 ± 0.3	4.3 ± 0.0	BP
*β*-CD	[−9, 6]	−5.3	28.1 ± 3.4	−1.5 ± 0.5	BS
*γ*-CD	−7	−5.0	32.7	−0.7	BS
*γ*-CD	[−6, 8]	−5.0	151.8 ± 6.5	1.2 ± 0.0	BP

*^a^* Using a value in this interval as the center Z of the docking search space enabled us to predict similar binding modes of [host:guest] complexes (Figure 2). *^b^* Binding affinities for [CD:DOPO] complexes. *^c^* Guest orientation relative to host (Figure 1b). *^d^* A value of 0.0 indicates that the standard deviations were smaller than 0.1. *^e^* Refer to Figure 1a for the definition of binding modes.

**Table 2:** Decomposition of [host:guest] binding free energies (kcal/mol) for cyclodextrin complexes with DOPO for BS and BP binding modes via the MM–PBSA analysis.

Energy		[*α*-CD:DOPO]		[*β*-CD:DOPO]		[*γ*-CD:DOPO]	
		BS	BP	BS	BP	BS	BP
MM	Δ*E*_bonded_	2.3 ± 0.3	2.8 ± 0.3	2.0 ± 0.3	1.9 ± 0.4	1.8 ± 0.3	1.3 ± 0.4
	Δ*E*_bonded,H_	2.0 ± 0.3	2.6 ± 0.3	1.8 ± 0.3	1.7 ± 0.3	1.6 ± 0.3	1.1 ± 0.3
	Δ*E*_bonded,G_	0.3 ± 0.1	0.3 ± 0.1	0.2 ± 0.1	0.2 ± 0.1	0.2 ± 0.1	0.2 ± 0.1
	Δ*E*_vdW_	−19.1 ± 0.1	−19.6 ± 0.1	−26.9 ± 0.2	−24.4 ± 0.1	−23.3 ± 0.9	−22.2 ± 0.4
	Δ*E*_elec_	−3.0 ± 0.4	−6.8 ± 0.2	−5.2 ± 0.3	−9.0 ± 0.5	−5.1 ± 1.0	−4.2 ± 0.5
	Δ*E*_nonbonded_	−22.1 ± 0.3	−26.4 ± 0.4	−32.1 ± 0.4	−33.4 ± 0.5	−28.3 ± 1.6	−26.4 ± 0.3
	Δ*E*_MM_	−19.9 ± 0.4	−23.5 ± 0.4	−30.1 ± 0.4	−31.5 ± 0.6	−26.6 ± 2.8	−25.1 ± 0.4
solvation	Δ*G*_polar_	12.4 ± 0.3	15.4 ± 0.2	18.6 ± 0.3	20.4 ± 0.4	17.1 ± 1.2	14.9 ± 0.2
	Δ*G*_nonpolar_	−1.7 ± 0.0	−1.8 ± 0.0	−2.3 ± 0.0	−2.2 ± 0.0	−2.3 ± 0.1	−2.3 ± 0.0
	Δ*G*_sol_	10.7 ± 0.3	13.6 ± 0.2	16.3 ± 0.3	18.1 ± 0.4	14.8 ± 1.1	12.7 ± 0.2
total	Δ*E*_bind_	−9.2 ± 0.4	−10.0 ± 0.3	−13.8 ± 0.3	−13.4 ± 0.4	−11.7 ± 0.4	−12.5 ± 0.4

Refer to Equations (1)–(3) for the energy decomposition. Block averaging was used to estimate the standard deviations for better statistics.

**Table 3:** Structural properties of isolated (free) host and [host:guest] complexes for the binding states of BS and BP modes in gas and water phases optimized at the B3LYP/6-31G** level of theory.

Host	State	Gas Phase				Water Phase			
		*τ* (Degree)	Circularity	*θ* (Degree)	COM Distance (Å)	*τ* (Degree)	Circularity	*θ* (Degree)	COM Distance (Å)
*α*-CD	crystal	85.2	0.950			85.2	0.950		
	free	78.8	0.949			78.4	0.938		
	BS	77.5	0.958	57.1	−5.2	77.7	0.933	31.1	−5.8
	BP	87.6	0.961	169.5	4.5	88.3	0.956	171.9	4.3
*β*-CD	crystal	84.5	0.935			84.5	0.935		
	free	81.7	0.955			80.9	0.941		
	BS	83.6	0.932	24.6	1.5	83.7	0.923	24.5	1.7
	BP	80.7	0.964	171.6	3.8	83.1	0.960	160.6	2.5
*γ*-CD	crystal	83.8	0.964			83.8	0.964		
	free	79.5	0.954			78.4	0.942		
	BS	81.8	0.912	23.1	−0.1	79.8	0.908	25.4	0.6
	BP	79.0	0.952	147.4	1.6	78.3	0.939	148.9	1.5

Crystal structures of cyclodextrins were extracted from the PDB database (PDB IDs: 1CXF for *α*-CD, 3CGT for *β*-CD, and 1D3C for *γ*-CD) and were given for comparison with QM calculations.

**Table 4:** Force field-based energy decomposition analysis (EDA-FF) of the QM-optimized [host:guest] complexes in gas and water phases for the binding modes of BS and BP.

Host	Charge Method	Optimized Complexes in Gas								Optimized Complexes in Water							
		Δ*E*_elec_		Δ*E*_rep_		Δ*E*_disp_		Δ*E*_FF_		Δ*E*_elec_		Δ*E*_rep_		Δ*E*_disp_		Δ*E*_FF_	
		BS	BP	BS	BP	BS	BP	BS	BP	BS	BP	BS	BP	BS	BP	BS	BP
*α*-CD	MK	−9.4	−3.4	8.4	16.3	−23.1	−32.7	−24.1	−19.8	−8.3	−3.0	6.6	14.4	−20.0	−31.6	−21.7	−20.2
	CHELPG	−9.4	−3.0					−24.1	−19.3	−8.4	−2.5					−21.8	−19.8
	RESP	−9.6	−3.7					−24.2	−20.1	−8.5	−3.0					−21.9	−20.2
	RESP2	−10.8	−4.4					−25.5	−20.8	−9.5	−3.6					−22.9	−20.8
	FFMD	−9.9	−2.8					−24.5	−19.2	−8.4	−1.8					−21.8	−19.1
*β*-CD	MK	−4.2	−10.7	19.8	10.6	−41.7	−27.1	−26.0	−27.3	−3.3	−10.6	17.8	9.8	−40.0	−26.4	−25.5	−27.1
	CHELPG	−3.6	−10.5					−25.5	−27.0	−2.8	−10.4					−25.1	−26.9
	RESP	−4.7	−10.4					−26.5	−26.9	−4.0	−10.2					−26.2	−26.8
	RESP2	−6.0	−12.9					−27.8	−29.4	−5.1	−12.6					−27.4	−29.2
	FFMD	−3.9	−11.4					−25.7	−27.9	−2.9	−11.3					−25.2	−27.9
*γ*-CD	MK	−4.5	−4.3	9.1	6.3	−27.8	−23.8	−23.2	−21.8	−3.2	−3.5	9.8	7.1	−26.4	−25.4	−21.6	−20.9
	CHELPG	−4.1	−4.1					−22.8	−21.6	−3.0	−3.4					−21.4	−20.7
	RESP	−4.0	−4.2					−22.7	−21.7	−3.3	−3.3					−21.7	−20.7
	RESP2	−5.5	−5.5					−24.2	−22.9	−4.6	−4.5					−23.0	−21.8
	FFMD	−6.2	−5.3					−24.9	−22.7	−3.8	−4.3					−22.2	−21.7

Atomic charges were derived with the electrostatic potential (ESP) fitting methods of Merz–Kollmann (MK), CHELPG, RESP, and RESP2. RESP is short for the restrained ESP; RESP2 was a variant of RESP and considered as the charge distribution in different media. FFMD indicates that atomic charges were extracted from the force field parameters used in our MD simulations (q4md-CD for host and GAFF2 for guest), in which the charge derivation followed the RESP protocol as well.

**Table 5:** Decomposition of binding free energies (kcal/mol) for [host:guest] complexes in the BS and BP binding modes via sobEDAw analysis.

Energies		Optimized Complexes in Gas						Optimized Complexes in Water					
		[*α*-CD:DOPO]		[*β*-CD:DOPO]		[*γ*-CD:DOPO]		[*α*-CD:DOPO]		[*β*-CD:DOPO]		[*γ*-CD:DOPO]	
		BS	BP	BS	BP	BS	BP	BS	BP	BS	BP	BS	BP
interaction energy	Δ*E*_els_	−17.3	−14.7	−19.6	−22.8	−12.9	−10.2	−14.3	−12.9	−17.3	−18.7	−9.9	−9.4
	Δ*E*_x_	−7.9	−10.4	−13.1	−9.9	−5.3	−2.4	−7.6	−8.8	−11.4	−8.4	−3.5	−2.5
	Δ*E*_rep_	40.1	51.4	64.1	47.2	33.0	22.4	33.9	46.4	58.7	45.1	26.3	22.9
	Δ*E*_xrep_	27.8	34.7	43.2	32.4	22.9	16.3	23.2	31.7	39.8	31.6	18.8	16.7
	Δ*E*_orb_	−9.2	−6.7	−8.7	−9.6	−5.3	−4.1	−7.5	−6.3	−8.1	−9.3	−4.5	−4.1
	Δ*E*_DFTc_	−11.1	−15.0	−18.8	−12.7	−11.4	−8.7	−8.1	−14.1	−17.8	−12.6	−9.6	−8.8
	Δ*E*_dc_	−19.2	−25.6	−32.5	−22.1	−22.5	−19.7	−13.7	−25.1	−31.8	−24.7	−21.3	−20.0
	Δ*E*_c_	−30.3	−40.6	−51.3	−34.8	−33.8	−28.4	−21.7	−39.2	−49.6	−37.3	−31.0	−28.8
	Δ*E*_disp_	−25.9	−34.3	−43.4	−29.9	−29.1	−24.7	−18.7	−33.3	−42.1	−32.1	−26.9	−25.1
	Δ*E*_int_	−24.7	−21.0	−28.5	−29.8	−24.4	−22.7	−17.3	−20.7	−27.8	−28.5	−22.5	−21.9
deformation effect	Δ*E*_def,H_	1.9	12.1	3.8	−0.3	3.8	0.5	1.3	14.0	3.7	5.5	0.7	0.0
	Δ*E*_def,G_	0.2	0.2	0.6	0.6	0.2	0.1	0.4	0.2	0.7	0.4	0.2	0.1
	Δ*E*_def_	2.1	12.3	4.4	0.3	3.9	0.6	1.8	14.2	4.4	5.9	0.9	0.1
solvation effect	Δ*E*_polar_	8.9	5.8	8.7	12.3	7.6	7.2	9.1	5.0	7.7	16.7	6.1	6.0
	Δ*E*_nonpolar_	−2.4	−2.4	−3.5	−2.6	−2.4	−1.9	−2.6	−2.3	−3.4	−3.9	−2.1	−1.8
	Δ*E*_sol_	6.5	3.4	5.2	9.7	5.2	5.3	6.5	2.7	4.3	12.8	4.0	4.2
thermodynamic correction	Δ*G*_corr_	17.6	15.0	19.2	20.8	16.9	16.7	16.9	14.3	18.6	19.8	16.8	16.4
total	Δ*G*_bind_	1.5	9.7	0.2	1.0	1.6	0.0	7.9	10.5	−0.6	10.0	−0.9	−1.1

Optimized isolated and [host:guest] complex structures in gas and water phases at B3LYP/6-31G(d,p) were used for the sobEDAw analysis. Energy decompositions of the interaction energies were calculated at the B3LYP-D3(BJ)/6-31+G(d,p) level of theory. Refer to Equations (5)–(9) for the decomposition.

## Data Availability

Data are contained within the article.

## Supplementary Materials

The following supporting information can be downloaded at: https://www.mdpi.com/article/10.3390/molecules29102244/s1, Table S1. Time averaged [host:guest] hydrogen bonds during MD simulations for cyclodextrins (CDs) complexes with DOPO in the binding modes of BS and BP. Figure S1: Distribution of [host:guest] distances; Figures S2–S7: [host:guest] distance and guest orientation for *α*-CD, *β*-CD, and *γ*-CD in the binding modes of BS and BP.
