# Supplementary material for: Computational Insights into Cyclodextrin Inclusion Complexes with the Organophosphorus Flame Retardant DOPO
Source: Molecules. 2024 May 10;29(10):2244. doi: 10.3390/molecules29102244 (PMC11124075; doi:10.3390/molecules29102244)
Supplement: Supplementary file 1 [file molecules-29-02244-s001.zip › molecules-2988461-supplementary.pdf]

Supplementary Materials for

Computational Insights into Cyclodextrin Inclusion Complexes with the Organophosphorus Flame Retardant DOPO

Le Ma, Yongguang Zhang, Puyu Zhang and Haiyang Zhang *

Department of Biological Science and Engineering, School of Chemistry and Biological Engineering, University of Science and Technology Beijing, Beijing 100083, China

* Correspondence: zhanghy@ustb.edu.cn

Table S1. Time averaged host–guest hydrogen bonds during MD simulations for cyclodextrins (CDs) complexes with DOPO in the binding modes of BS and BP.

Cyclodextrin	BS	BP
α-CD	0.2 ± 0.4	0.6 ± 0.5
β-CD	0.1 ± 0.2	0.6 ± 0.5
γ-CD	0.1 ± 0.3	0.5 ± 0.5

MD trajectories for the MM-PBSA analysis were used to calculate the hydrogen bonds between host and guest molecules.

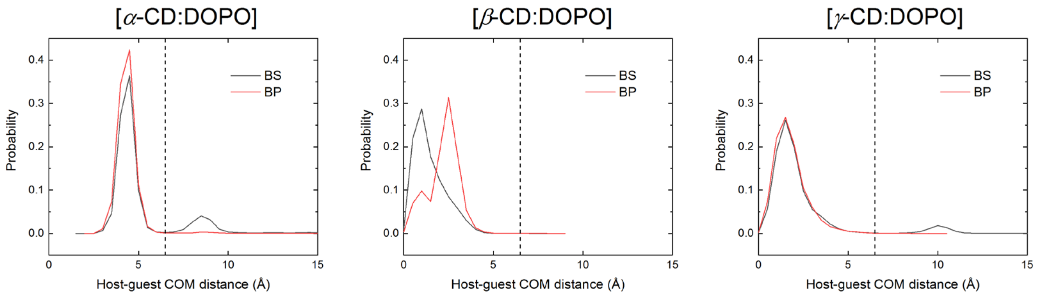

Figure S1. Distribution of the center of mass (COM) distances between host and guest for [α-CD:DOPO], [β-CD:DOPO], and [γ-CD:DOPO] complexes during 100 ns MD simulations using BS and BP binding modes (Figure 1a) as initial configurations. The dash lines indicated a distance of 6.5 Å, which separated the binding pose of guest included in the CD cavity from the pose of guest locating outside the CD cavity.

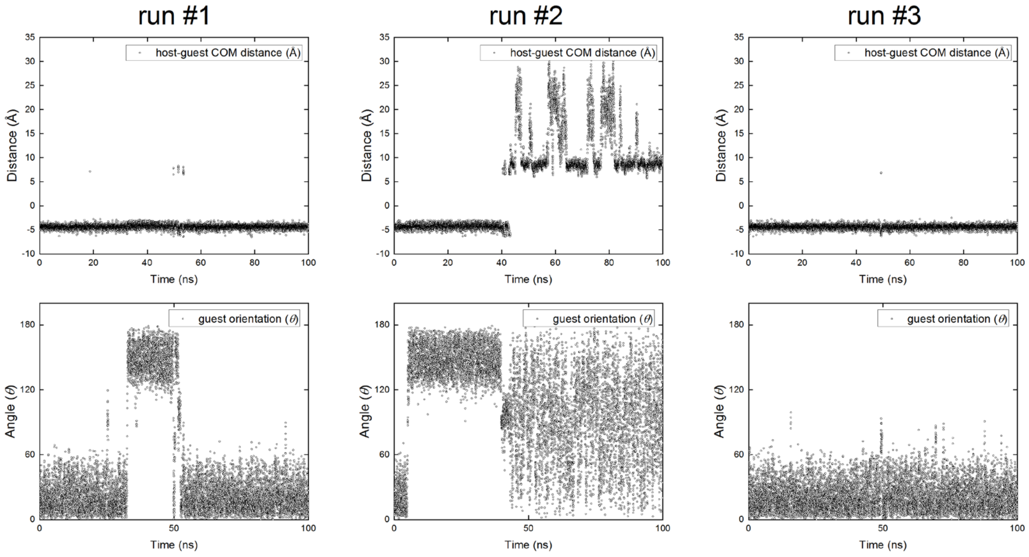

Figure S2. Host–guest COM distance (top) and guest orientation (θ, bottom) for [α-CD:DOPO] complexes during 100 ns simulations using the BS binding mode (Figure 1a) as initial configurations. Each simulation was repeated three times (runs #1, #2, and #3) with different initial velocities. A negative value for the distance indicated that the COM of guest was much closer to the secondary rim (S-rim) of CDs than the primary rim (P-rim); a positive value meant the guest COM was much closer to the P-rim (Figure 1). A distance of > 6.5 Å indicated that the guest was escaped from the CD cavity.

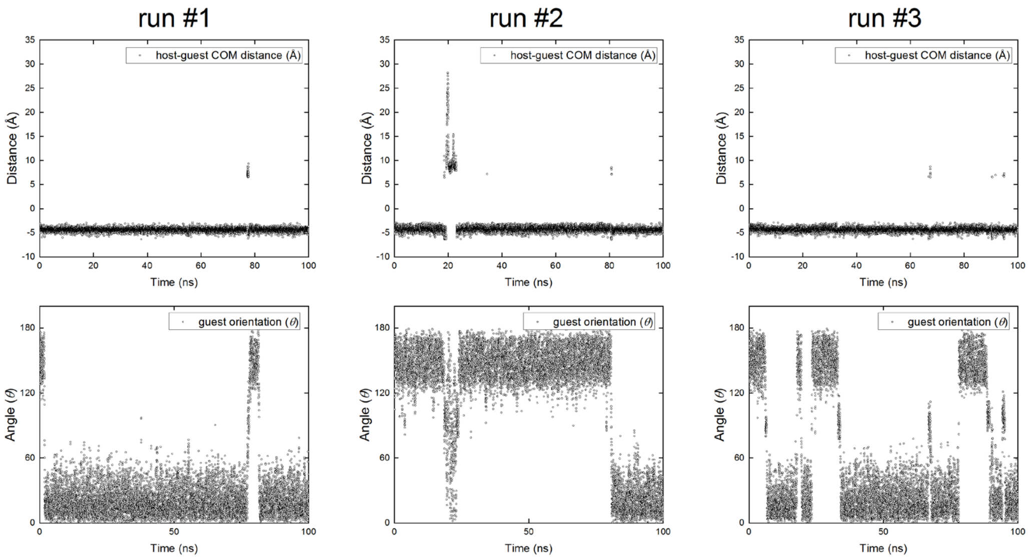

Figure S3. Host–guest COM distance (top) and guest orientation (θ, bottom) for [α-CD:DOPO] complexes during 100 ns simulations using the BP binding mode (Figure 1a) as initial configurations. Each simulation was repeated three times (runs #1, #2, and #3) with different initial velocities. A negative value for the distance indicated that the COM of guest was much closer to the secondary rim (S-rim) of CDs than the primary rim (P-rim); a positive value meant the guest COM was much closer to the P-rim (Figure 1). A distance of > 6.5 Å indicated that the guest was escaped from the CD cavity.

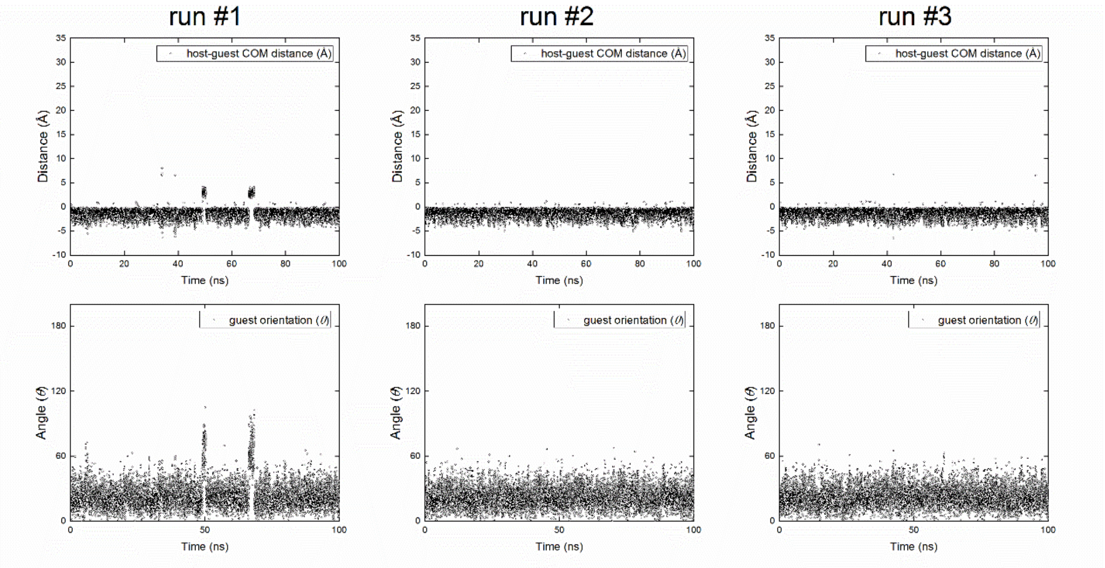

Figure S4. Host–guest COM distance (top) and guest orientation (θ, bottom) for [β-CD:DOPO] complexes during 100 ns simulations using the BS binding mode (Figure 1a) as initial configurations. Each simulation was repeated three times (runs #1, #2, and #3) with different initial velocities. A negative value for the distance indicated that the COM of guest was much closer to the secondary rim (S-rim) of CDs than the primary rim (P-rim); a positive value meant the guest COM was much closer to the P-rim (Figure 1). A distance of > 6.5 Å indicated that the guest was escaped from the CD cavity.

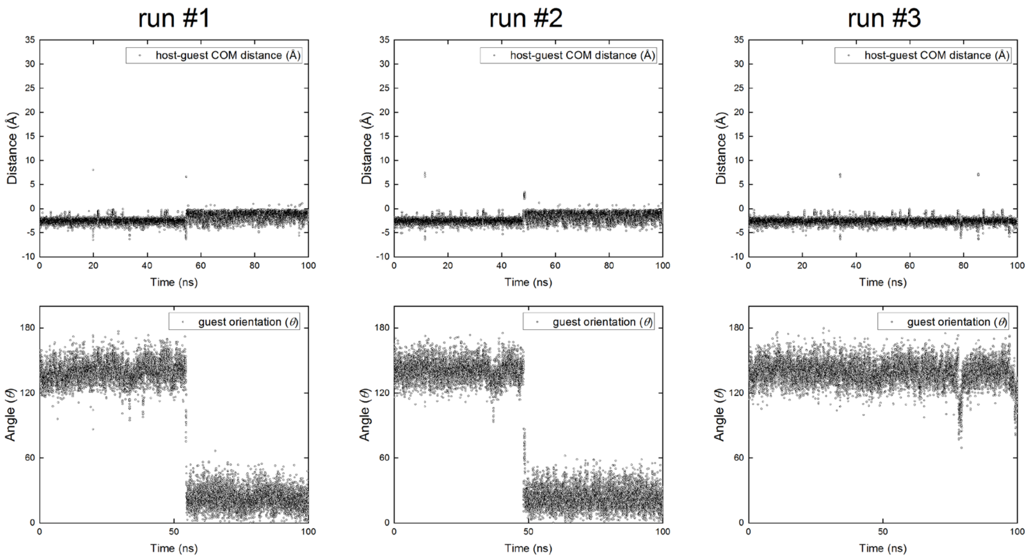

Figure S5. Host–guest COM distance (top) and guest orientation (θ, bottom) for [β-CD:DOPO] complexes during 100 ns simulations using the BP binding mode (Figure 1a) as initial configurations. Each simulation was repeated three times (runs #1, #2, and #3) with different initial velocities. A negative value for the distance indicated that the COM of guest was much closer to the secondary rim (S-rim) of CDs than the primary rim (P-rim); a positive value meant the guest COM was much closer to the P-rim (Figure 1). A distance of > 6.5 Å indicated that the guest was escaped from the CD cavity.

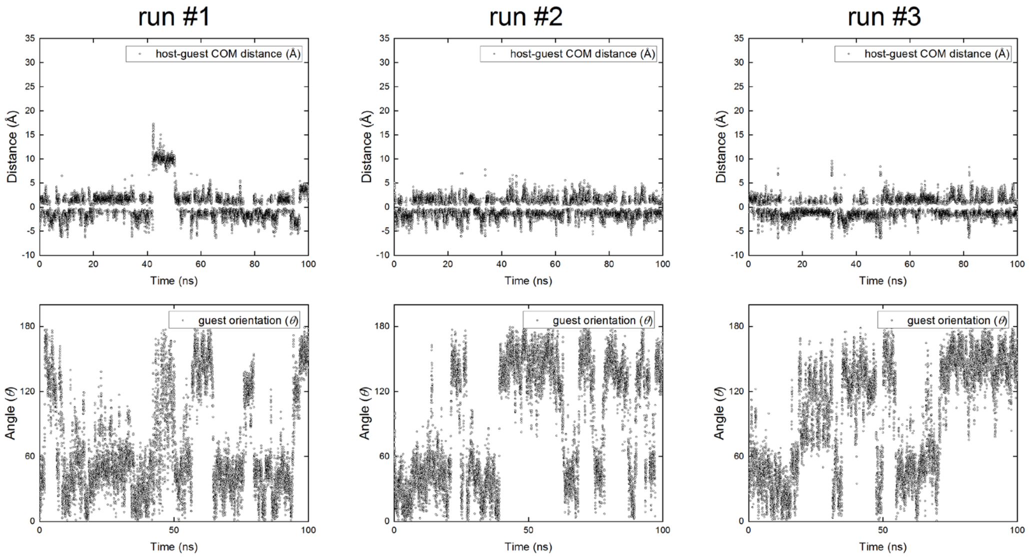

Figure S6. Host–guest COM distance (top) and guest orientation (θ, bottom) for [γ-CD:DOPO] complexes during 100 ns simulations using the BS binding mode (Figure 1a) as initial configurations. Each simulation was repeated three times (runs #1, #2, and #3) with different initial velocities. A negative value for the distance indicated that the COM of guest was much closer to the secondary rim (S-rim) of CDs than the primary rim (P-rim); a positive value meant the guest COM was much closer to the P-rim (Figure 1). A distance of > 6.5 Å indicated that the guest was escaped from the CD cavity.

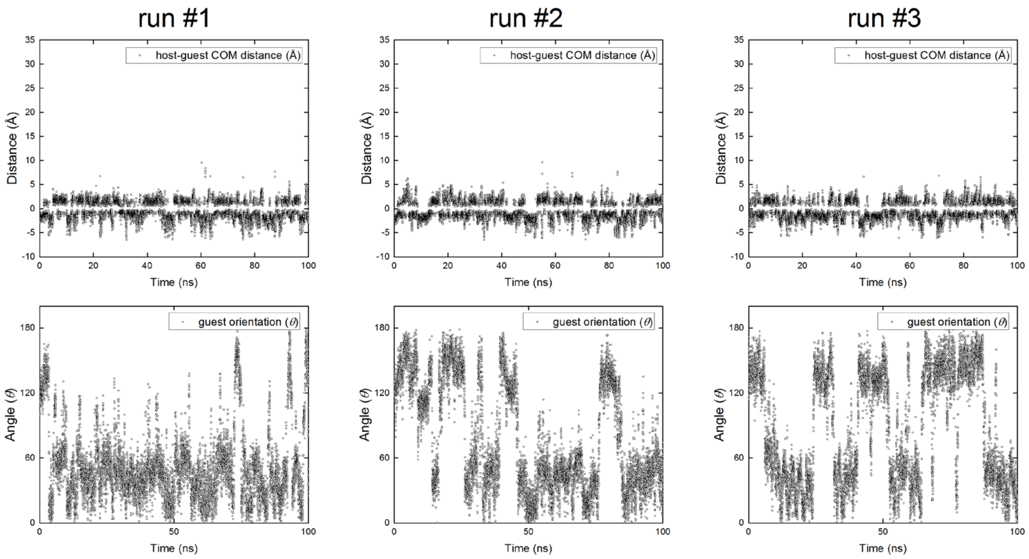

Figure S7. Host–guest COM distance (top) and guest orientation (θ, bottom) for [γ-CD:DOPO] complexes during 100 ns simulations using the BP binding mode (Figure 1a) as initial configurations. Each simulation was repeated three times (runs #1, #2, and #3) with different initial velocities. A negative value for the distance indicated that the COM of guest was much closer to the secondary rim (S-rim) of CDs than the primary rim (P-rim); a positive value meant the guest COM was much closer to the P-rim (Figure 1). A distance of > 6.5 Å indicated that the guest was escaped from the CD cavity.
